# Emergence and expansion of dengue in Paltas: possible implications of the COVID-19 pandemic and climatic variations

**DOI:** 10.1186/s41182-025-00689-6

**Published:** 2025-01-29

**Authors:** Beatriz Quintero, Angélica X. Ramón-Ochoa, Solbey Morillo-Puente, Daniel A. Tenezaca-Ramón, Alejandra S. Cevallos-Naranjo

**Affiliations:** 1 School of Medicine, Private Technical University of Loja, Loja, 110101 Ecuador; 2 Doctor of Medicine and Surgery, Zonal Management of Health Surveillance, Prevention and Control, Loja, Ecuador; 3 Department of Methodology, Faculty of Legal and Political Sciences, Los Andes University (ULA), Merida, Venezuela; 4 School of Business Administration, Sabal University, Miami, FL USA

**Keywords:** Dengue, Arbovirus, Climate change, Pandemics, COVID-19

## Abstract

**Introduction:**

Dengue is one of the most widespread arboviruses in Latin America and is now affecting areas previously free of transmission. The COVID-19 pandemic and climatic variations appear to have affected the incidence of the disease, abundance of vectors and health programs related to dengue in some countries.

**Objective:**

To analyze the epidemiology of dengue in Paltas, Ecuador (2016–2022), compare the periods before and during the COVID-19 pandemic, examine entomological reports and discuss the possible implications of the COVID-19 pandemic and climatic variations.

**Methodology:**

In this observational, retrospective study, cases of dengue registered in the SIVE-Alert epidemiological surveillance system and the available larval indices were examined.

**Results:**

No autochthonous cases were reported before 2016. Between 2016 and 2022, 182 cases without warning signs were reported, mostly in women (51.1%), people ≥ 20 years (68.7%) and people living in urban areas (78.6%). During the COVID-19 pandemic, there was a significant decline in cases in urban areas, with displacement toward rural areas (*p* < 0.001). A clear pattern of dengue incidence was observed throughout the year, with a predominance (84.6%) in epidemiological weeks 16–39 (April–September), which coincided with the dry season in the region. In 2016 and 2018, larval rates were high in urban areas but decreased in 2019. Postpandemic incidence rates increased in urban and rural areas, even in areas without transmission of the disease.

**Conclusions:**

There is a clear pattern of incidence of dengue in the dry season in the region. After the 2016 outbreak, larval cases and rates decreased, suggesting the effectiveness of vector control before the COVID-19 pandemic. However, during the pandemic there was a resurgence in dengue with expansion in rural and urban areas. The increase in larval rates during the COVID-19 pandemic, even in dengue-free areas, is worrisome. A critical challenge in the control of mosquito breeding sites is climatic variations, which increase the need to reserve water for domestic use.

## Introduction

Vector-borne diseases are a global challenge, representing more than 17% of all infectious diseases and more than 700,000 deaths annually; among them, dengue is as the most common and most widespread arbovirus transmitted by *Aedes* spp. mosquitoes [1, 2]. The main vector for dengue transmission is *Aedes aegypti*, and *Ae. albopictus* is considered a secondary vector [3]. In the Americas, the incidence of dengue has increased alarmingly in recent years, from 1.5 million in 1980–1989 to 17.5 million in 2010–2019. More than 3.18 million cases were registered in 2019, the highest annual number of cases historically reported. During the COVID-19 pandemic period (2020–2022), the number of dengue cases slightly decreased, while during 2023, 4.1 million cases were reported, surpassing the previous highest figure. Dengue currently affects 42 countries and territories in the region, with outbreaks occurring in 15 countries [4].

The recent proliferation of viral diseases transmitted by vectors, such as dengue, has been associated with factors such as global trade, travel, demographic expansion, urbanization and climate change [5]. Climate change is considered one of the most worrisome threats to public health and is associated with the transmission, appearance and reappearance of infectious diseases, as well as the survival, propagation and reproduction of their respective vectors [6, 7]. In the twenty-first century, an increase in temperature between 1.5 and 5.8 °C has been reported worldwide, which is correlated with an increase in the incidence of multiple infectious diseases sensitive to climate, such as dengue [8]. The COVID-19 pandemic has exacerbated this situation, causing interruptions in the prevention and treatment of dengue, as well as variations in the incidence of the disease [9, 10]. Due to the increasing incidence and geographic expansion of dengue worldwide, it is essential to investigate its appearance in areas without prior autochthonous transmission, such as the Paltas canton where no cases were recorded before 2016. In this study, the epidemiology of dengue in the Paltas canton, Province of Loja, Ecuador, from 2016 to 2022 and the differences between the periods before and during the COVID-19 pandemic in the context of climate change were analyzed. Additionally, larval indices are discussed as indicators of vector control and an approximation of the possible expansion of the dengue vector in the area.

## Methodology

### Characteristics of the study area

Paltas is one of the 16 cantons of the Province of Loja, Ecuador. It is divided into 9 parishes: 2 urban parishes, Catacocha and Lourdes, and 7 rural parishes, Cangonamá, Casanga, Guachanamá, Lauro Guerrero, Orianga, San Antonio and Yamana. According to the National Institute of Statistics and Censuses (INEC), the population projection for this canton in 2020 was 23,471 inhabitants. In Paltas, the main agricultural activities are as follows: corn, coffee, artisanal sugarcane, and short-cycle crops; livestock operations; and forest plantations, among others. The region has an area of 1,124 km^2^, and the altitude ranges from 400 m above sea level (MASL)to 3,087 MASL. Paltas experiences two types of climates: a dry megathermal tropical climate (Aw) in the lower part of the canton (48,877.31 ha) and a semi-humid mesothermal equatorial climate (Cfb) in the higher part of the canton (67,039.16 ha). Rainfall in the region ranges from 600 to 1500 mm; June–September are the driest months (3 mm), and January–April are the months with the highest rainfall (232 mm). The average annual temperature is 16.8 °C; September is the hottest month (average temperature, 19.1 °C), and January to March are the coldest months (average temperature, 17.8 °C). Paltas has a serious deficit of water for consumption and agricultural activities that affects urban and rural areas [11].

### Research design, ethical aspects and data analysis.

In this observational, cross-sectional retrospective study, all dengue cases registered between 2016 and 2022 in the National Public Health Surveillance System of Ecuador through the ViEpi platform were reviewed with the permission of the respective institutions. No records were excluded since the required information was available for all cases. In addition, all the entomological reports (larval indices) in the epidemiology department of the region were analyzed, specifically the larval indices corresponding to the years 2016, 2018 and 2019. Notably, there was no entomological data for some of the years included in the study period, which represents a limitation in evaluating the vector control strategies implemented. For this reason, an additional analysis was carried out using the larval indices available for 2023, with the aim of providing a more recent approach for vector control and analyzing the possible spread of dengue in the region. In the interpretation of the larval indices, a house index (HI) < 5%, Breteau index (BI) < 5, and container index (CI) < 10% were used as indicators of protection against epidemics caused by dengue fever and the need for greater vector control to avoid epidemics [12]. The study was performed with institutional permission and with the approval of the Ethics Committee of the Private Technical University of Loja (UTPL), Ecuador (code UTPL-CEISH-2022-PG07). The characteristics available in the registry were dengue severity, age group and sex, stratified by epidemiological week, year and parish of notification. Descriptive data are reported as frequencies and percentages.

Owing to the categorical nature of the variables, comparisons of dengue cases before the COVID-19 pandemic (2016–2019) and during the pandemic (2020–2022) with respect to sex, age, and geographical area were primarily conducted using Chi-square test. However, Fisher's exact test was applied in cases where the sample size within specific groups was less than 5, to ensure compliance with statistical assumptions. All statistical tests were performed at a significance level of 95%.

## Results

**Fig. 1:**
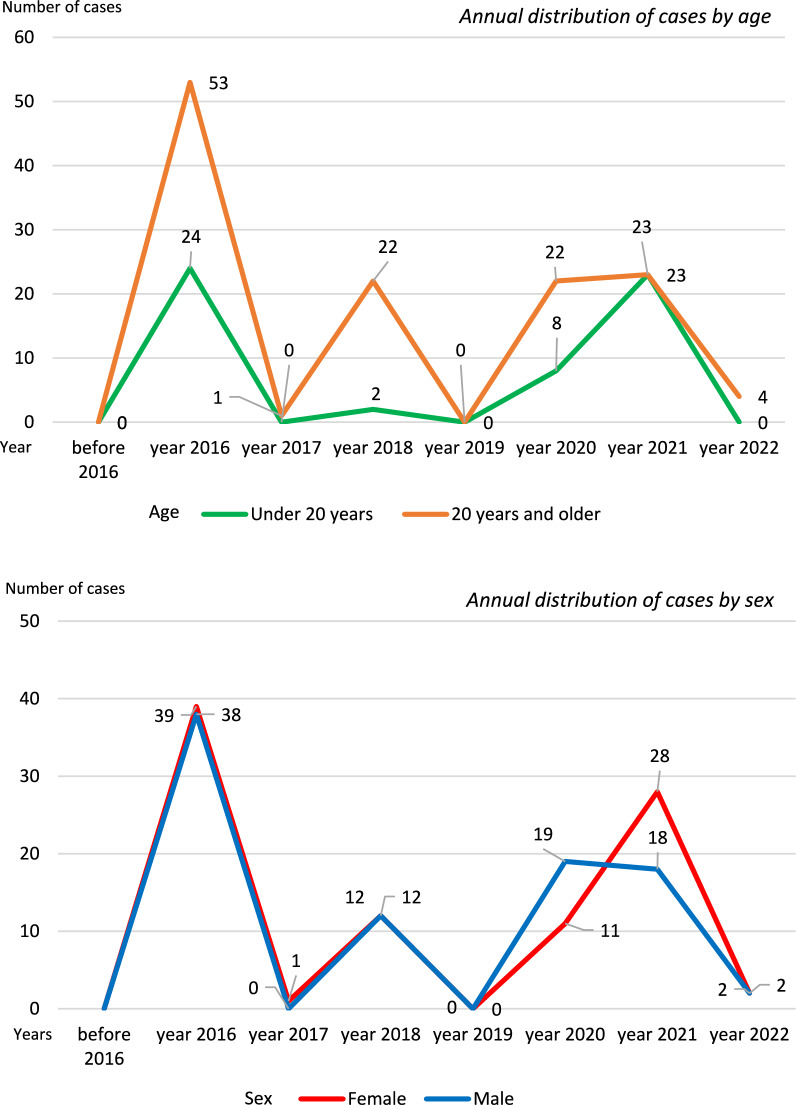
Annual distribution of dengue cases according to age and sex in Paltas, 2016–2022

A total of 182 cases of dengue were reported between 2016 and 2022, all of which were cases without warning signs. In Paltas, dengue fever was not reported until 2016. During the first years of its appearance, the number of cases was minimal; incidence rates decreased in 2017 (one case) and 2019 (no cases), but there was a resurgence of cases during the COVID-19 pandemic, particularly between 2020 and 2021 (Fig. 1). From 2016 to 2022, dengue cases occurred predominantly in female patients (51.1%) and those aged ≥ 20 years (68.7%) (Fig. 1), although there were no significant differences between the periods before and during the COVID-19 pandemic (Table 1).

**Table 1:** Distribution of dengue cases according to sex, age and parish before and during COVID-19 in Paltas, 2016–2022

Characteristic	Before COVID-19 (2016–2019) N = 102 (annual x̅, 25.5)		During COVID-19 (2020–2022) N = 80 (annual x̅, 26.6)		Total (2016–2022) N = 182		*p*
	#	%^a^	#	%^b^	#	%^c^	
Sex							
Male	50	49.0	39	48.7	89	48.9	0.963^d^
Female	52	50.9	41	51.2	93	51.1	
Age							
Under 20 years	26	25.5	31	38.7	57	31.3	0.055^e^
< 15	14	13.7	17	21.2			
15–19	12	11.8	14	17.5			
20 years and older	76	74.5	49	61.3	125	68.7	
20–64	55	53.9	41	51.2			
65 and older	21	20.5	8	10			
Parishes							
Urban	102	100.0	41	51.2	143	78.6	0.000^f^
Rural	0	0	39	48.8	39	21.4	

^a^Percentage of the pre-pandemic total

^b^Percentage of the post-pandemic total

^c^Percentage of the 2016–2022 total

^d^*P* value obtained with the chi-square test of independence at a significance level of 95% by comparing the periods before and during the COVID-19 pandemic according to sex (male vs. female)

^e^*P* value obtained with the chi-square test of independence at a significance level of 95% by comparing the periods before and during the COVID-19 pandemic according to age (< 20 years vs. ≥ 20 years)

^f^*P* value obtained using Fisher's exact test at a significance level of 95% by comparing the periods before and during the COVID-19 pandemic according to parish (urban vs. rural)

To date, cases have been reported in 4 of the 9 parishes of the canton: 2 urban parishes, Lourdes and Catacocha, and 2 rural parishes, Casanga and Guachanamá. Over the years, a predominance of cases was observed in urban areas (78.6%), with 4 urban cases occurring for each case in rural areas (Fig. 1). Notably, before the COVID-19 pandemic, cases were restricted to urban areas and limited to a single parish per year. As of 2020, geographical dispersion was observed, with cases appearing in several parishes simultaneously in both rural and urban areas. In fact, when comparing the period before the COVID-19 pandemic with the period during the pandemic, a statistically significant decline in cases was observed in the urban areas, with the displacement of cases to the rural areas (*p* < 0.001) (Table 1).

**Fig. 2:**
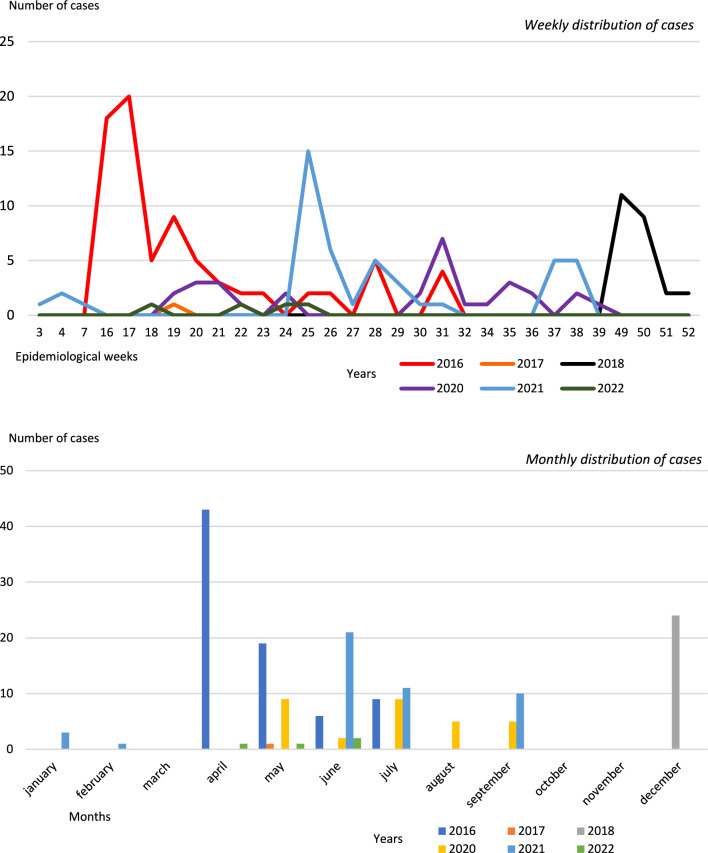
Distribution of dengue cases according to incidence by epidemiological week and month in Paltas, 2016–2022

On another note, a clear pattern of dengue incidence was observed according to the epidemiological week of notification, with the majority of cases (N = 154/182; 84.6%) occurring between epidemiological weeks 16 and 39, i.e., between April and September (Fig. 2).

**Fig. 3:**
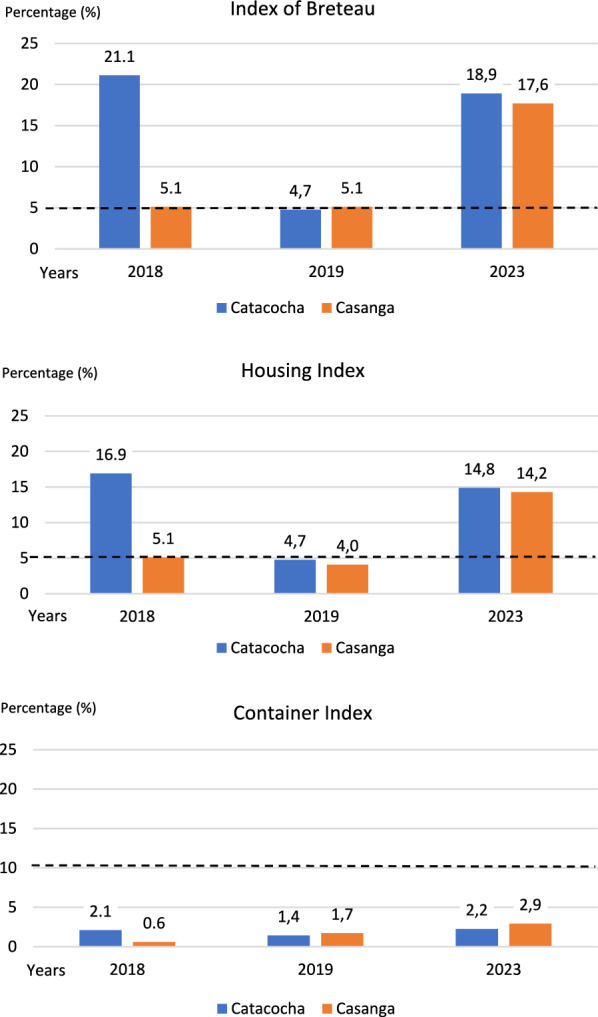
Larval indices of the Catacocha and Casanga parishes, Paltas canton, 2016–2023

On the other hand, the larval indices remained higher in urban areas than in rural areas (Fig. 3). For 2016, HI reports were available only for Catacocha (100%) and Casanga (57.1%) (data not shown). In 2018, in Catacocha, BI and HI were high and decreased to close to 5% in 2019. In contrast, these indices remained low in Casanga until 2019. However, by 2023, the BI and HI increased again in both parishes. In contrast, the CI values never exceeded 10% in any of the parishes. Notably, in 2023, entomological reports were also obtained from other parishes of the canton that have not yet been affected by dengue. These reports showed BI and HI values above 5%, higher than those reported in regions where dengue is common. For example, the BI was between 18.1 and 40.3 in Yámana and 14.8 in San Antonio. The HI was between 14.9 and 18.1 in Yámana and 10.8 in San Antonio (data not shown).

## Discussion

In the Paltas canton, a dengue-free area until 2016, dengue was more common in urban areas and between April and September. Over time, there were annual fluctuations in both the incidence of cases and the larval indices. There are concerns about the resurgence of cases and the geographical expansion of dengue during the COVID-19 pandemic.

Given the resurgence of cases and larval indices and the geographical expansion of dengue in Paltas after the pandemic, we must consider that the management, control and treatment of diseases transmitted by arthropods could have been delayed by the pandemic [10]. The pandemic has had a significant effect on the incidence of and health services related to dengue. In the Americas, during the first 6 months of 2020, a 10% decrease in dengue, chikungunya and Zika cases was reported versus the same period in 2019. Most cases occurred in Brazil, Paraguay, Bolivia, Argentina and Colombia [13]. A systematic review of cases in Southeast Asia revealed a significant disruption in the prevention, diagnosis, treatment, and management of tuberculosis, HIV, and dengue during the COVID-19 pandemic [9]. Studies conducted in Sri Lanka and Bhutan revealed a reduction in the provision of dengue-related health services. However, in these countries, there was also a reduction in the number of dengue cases reported during the pandemic, attributed in part to limited mobility and isolation measures [9]. The apparent decrease in dengue cases or their notification during the first years of the COVID-19 pandemic has been attributed to the implementation of public health measures, especially restrictions on human mobility (community quarantines, school closings and confinements). In addition, health services in response to the pandemic were prioritized, and health services for the diagnosis and surveillance of dengue may have been reduced, resulting in an underregistration of cases. The refusal of patients to visit health centers due to the fear of contracting COVID-19 or to avoid viral detection tests could also have had an influence [14].

However, in some countries, the COVID-19 pandemic was associated with an increase in dengue. According to the WHO, after the decrease in the notification of dengue cases in the first years of the pandemic (2020–2022), a global rebound has been observed, which has spread to regions previously free of dengue [4]. According to data from the Pan American Health Organization (PAHO), dengue incidence rates in the Americas showed considerable variability between 2016 and 2022. Before the COVID-19 pandemic (2016–2019), the highest incidence was recorded in 2019 (325.93 cases per 100,000 inhabitants), more than three times the rate observed in 2018 (77.51 cases per 100,000 inhabitants). In 2020, during the first year of the pandemic, the incidence dropped to 238.87 cases per 100,000 inhabitants. This decline may reflect the effects of strict public health measures, such as mobility restrictions and quarantines, which likely reduced mosquito exposure or hindered case detection and reporting. However, by 2022, dengue incidence rebounded to 283.58 cases per 100,000 inhabitants, aligning with the global pattern of dengue resurgence as pandemic-related restrictions eased. This trend highlights the intricate relationship between the COVID-19 pandemic and vector-borne diseases, underscoring the importance of resilient dengue surveillance and control systems during concurrent health crises [15].

In Ecuador, dengue incidence rates showed significant fluctuations during the study period. Before the pandemic (2016–2019), the incidence remained relatively stable, with a notable low in 2018 (18.38 cases per 100,000 inhabitants) and a moderate increase in 2019 (49.91 cases per 100,000 inhabitants). The onset of the COVID-19 pandemic brought a sharp rise in dengue incidence, peaking at 118.31 cases per 100,000 inhabitants in 2020. Although there was a slight decrease in 2021 and 2022, the incidence remained consistently higher than pre-pandemic levels [15]. In Paltas, a region that had been free of dengue prior to 2016, the pandemic coincided with a resurgence and geographic expansion of the disease, similar to what occurred across Ecuador. This could reflect the broader challenges faced throughout Ecuador in sustaining effective vector control measures during the pandemic. Factors such as climatic variations and changes in human activity likely played a role in the spread of dengue, underscoring the importance of strengthening public health strategies, not just in Paltas but across the country, to address both the immediate and long-term impacts of vector-borne diseases.

A review of data from 22 Asian and Latin American countries revealed that the incidence of dengue decreased significantly (16%; 2.73 million versus 2.29 million) in 2020–2021 compared with the average incidence in the 5 pre-pandemic years (2015–2019) [16]. However, in Brazil, Peru, Bolivia, Ecuador, Paraguay, Argentina, and Singapore, a higher incidence of dengue was reported during 2020 than before the pandemic. An increase in cases was found in Bangladesh (19-fold), Pakistan (sevenfold) and India (threefold) during the second year of the COVID-19 pandemic (2021). It is unclear whether the strict social measures implemented in response to the pandemic or underreporting due to the overload of resources caused by its management is related to the decrease in dengue cases reported during the pandemic [16].

To estimate infestation by *Aedes spp*. in a geographic area, the most commonly used surveillance methods are the larval indices, HI, BI and CI. The observations of yellow fever epidemics in urban areas suggest that values of HI < 5%, BI < 5 and CI < 10% provide protection against epidemics caused by other arboviruses, such as dengue, chikungunya and Zika. However, these thresholds for larval indices are not completely reliable in relation to the transmission of dengue virus, with the sampling of adult vectors being more reliable [17]. Despite these limitations, larval indices remain the main surveillance tool used in many *Aedes spp*. control programs, owing to their practicality and reproducibility. The Pan-American Health Organization (PAHO) recognizes the advantages of the larval indices, namely, a relatively small sample size (100–200 households), acceptable cost and no need for specialized personnel. In addition, these indices enable a focus on larval control efforts during the management or elimination of hatcheries; evaluation of the effectiveness of these measures, especially in difficult-to-access hatcheries; and guidance of educational messages addressed to the community [17]. Currently, larval indices, when correctly interpreted, are considered useful tools for determining the risk of dengue outbreaks and directing specific vector control actions. In this context, when the BI is similar to or equal to the HI, the problem is considered to be generalized. On the other hand, a BI much higher than the HI could indicate a focused problem, suggesting the implementation of specific control measures in that sector [18]. In the measurements taken during the study period and in 2023 in the Paltas canton, the BI was always greater than the HI, which indicates a localized problem that requires control measures focused on the affected sector.

Vector control continues to be the main method for the control and prevention of dengue. To achieve control, mosquito breeding sites, especially water reservoirs, should be eliminated; communities should be informed and encouraged to participate in dengue control; and active and continuous monitoring of the abundance of mosquitoes should be performed [3, 19, 20]. In Ecuador, during the 1950s, dengue and mosquitoes were eradicated with insecticides. However, failures in vector control and rapid unplanned urbanization favored the resurgence of dengue in 1989 [21]. Currently, dengue is endemic, with the common serotypes DENV-1 and DENV-2. By 2023, 26,847 cases were reported, with more than 80% of notifications occurring in 7 of the 24 provinces of Ecuador: Manabi, Santo Domingo, Guayas, Esmeraldas, Morona Santiago, El Oro and Napo [22]. Unlike the results in Paltas, the number of cases in the country overall were greater in those under 20 years of age (57.3%) and in men (50.7%). Importantly, *Ae. albopictus*, which was first reported in Guayaquil, Ecuador, in 2017, has already been identified in the northeastern lowlands and the Amazon basin [23]. To date, there are no reports on the presence of *Ae. albopictus* in Paltas. However, the establishment of this species of mosquito and climate change could increase the risk of dengue in other areas of Ecuador [23].

A critical point in the control of mosquito breeding sites in the canton seems to be the annual climatic variations associated with the need for water reservoirs for domestic use. The incidence of dengue is greater between April and September, when droughts and water restrictions occur, which could encourage the creation of water reservoirs. In various regions of the world, a strong association between the lack of public water supply and the incidence of dengue or *Aedes spp*. abundance. Poor or poorly planned urban development could result in an inadequate water supply, promoting the use of reservoirs and favoring mosquito breeding sites [24]. In some regions of Ecuador, such as Huaquillas and Guayaquil, an increase in the abundance of *Ae. aegypti* has been observed after a period of abundant rainfall or due to the interruption of running water services [25, 26].

Climate change has had positive and negative effects on the incidence of dengue. For example, the increase in dengue incidence in China was related to the temperature increase, whereas in Singapore, the heat surges were related to a decrease in the transmission of dengue fever [27, 28]. However, meteorological factors do not directly influence the incidence of dengue but rather have a direct effect on the vectors. These effects include larval/adult development and survival, duration of the gonotrophic cycle (host search and blood intake), reproductive behavior of the vector, extrinsic incubation periods of the virus, and dengue transmission rates [29].

At the global level, climate change affects the density or reproduction of *Aedes* spp. The factors that favor these changes include increases in temperature [30], extreme heat waves [27, 31, 32], excess water (number of rain events or floods) [27, 31–33] and dry spells [32]. In Kenya, the increased reproduction of *Ae. aegypti* in rural areas compared with that in urban areas was related to differences in the number of wells or puddles that formed after rain [32].

Human behaviors during climatic changes, such as the use of air conditioners, staying outside longer and especially the method of water storage, can modify the impact of the climate on vectors. For example, in Pakistan, a greater number of rain events from 2011 to 2014 increased the density of *Ae. albopictus*. However, in times of higher temperatures and less rain, the number of mosquitoes decreased, but they were not scarce due to the presence of artificial water reservoirs [31]. In Singapore, during times of extreme heat, people spend more time in air-conditioned environments, decreasing vector exposure and transmission [28]. In Kenya, out-of-home transmission prevails owing to living conditions and climate, especially because children spend a long time playing outdoors [32].

Finally, the experience of the Paltas canton, transforming from a dengue-free zone to an area of resurgence during the COVID-19 pandemic, highlights the need for a multifactorial approach to dengue control that considers the implications of climate change, changes in human behaviors, and major health phenomena such as the COVID-19 pandemic to ensure a more effective and sustainable response over time.

## Limitations

This study has several limitations that should be acknowledged. First, its retrospective nature prevents causal inferences from being made. Second, larval indices were not available for most years in the study period, which restricted an exhaustive analysis of vector control efforts in the area. Additionally, this research did not aim to analyze climate data, and as such, the study lacks accurate conclusions about the role of climate change in the spread of dengue. Another important limitation is the absence of data on COVID-19 cases and their potential relationship with the distribution of dengue cases, as well as the lack of more recent data on dengue. While such data would provide a clearer understanding of the pandemic's impact on the epidemiology of dengue in the region, their inclusion was not part of the original research design. The initial project did not intend to include COVID-19 cases or additional years beyond the approved timeframe. Therefore, this publication is limited to the data authorized by the Ministry of Health and ethics committee's approval. Finally, although calculating odds ratios could provide a more detailed approximation of the relationships between the variables under study, this was not possible due to the nature of the data provided by the Ministry of Health. The data were aggregated and pre-categorized, meaning individual-level data, including specific combinations of variables for each subject, were unavailable. This limitation prevents the calculation of odds ratios, which require access to individual-level data for accurate analysis.

## Conclusions

Dengue has experienced a resurgence and geographic expansion during the COVID-19 pandemic in the Paltas canton. Although vector control measures appeared to have controlled the problem initially, several factors, such as climatic variations and their consequences on changes in the behavior of the population, could impact the effectiveness of these sanitary measures. The canton offers space for research on the effects of climate change and community participation, which can help explain the reasons for the establishment, maintenance and spread of dengue in an area previously free of autochthonous transmission.

## Data Availability

No datasets were generated or analysed during the current study.
